# The Natural Flavone Acacetin Confers Cardiomyocyte Protection Against Hypoxia/Reoxygenation Injury via AMPK-Mediated Activation of Nrf2 Signaling Pathway

**DOI:** 10.3389/fphar.2018.00497

**Published:** 2018-05-15

**Authors:** Wei-Yin Wu, Yun-Da Li, Yu-Kai Cui, Chan Wu, Yi-Xiang Hong, Gang Li, Yao Wu, Ling-Jun Jie, Yan Wang, Gui-Rong Li

**Affiliations:** Xiamen Cardiovascular Hospital, Xiamen University, Xiamen, China

**Keywords:** acacetin, hypoxia-reoxygenation, cardioprotection, Nrf2, AMPK

## Abstract

The present study investigates the potential signal pathway of acacetin in cardioprotection against ischemia/reperfusion injury using an *in vitro* hypoxia/reoxygenation model in primary cultured neonatal rat cardiomyocytes and H9C2 cardiomyoblasts. It was found that acacetin (0.3–3 μM) significantly decreased the apoptosis and reactive oxygen species production induced by hypoxia/reoxygenation injury in cardiomyocytes and H9C2 cardiomyoblasts via reducing the pro-apoptotic proteins Bax and cleaved-caspase-3 and increasing the anti-apoptotic protein Bcl-2. In addition, acacetin not only suppressed the release of pro-inflammatory cytokines TLR-4 and IL-6 induced by hypoxia/reoxygenation injury, but also increased the secretion of anti-inflammatory cytokine IL-10. Moreover, acacetin increased Nrf2 and HO-1 in a concentration-dependent manner, and rescued SOD1 and SOD2 reduction induced by hypoxia/reoxygenation insult. These beneficial effects of acacetin disappeared in cells with silenced Nrf2, suggesting that Nrf2 activation participates in the cardioprotective effect of acacetin against hypoxia/reoxygenation insult. However, acacetin-induced Nrf2 activation was not observed in cells with silenced AMPK and in ventricular tissues of rat hearts treated with the AMPK inhibitor Compound C and subjected to ischemia/reperfusion injury. Our results demonstrate for the first time that AMPK-mediated Nrf2 activation is involved in the cardiomyocytes protection of acacetin against hypoxia/reoxygenation injury by activating a series of intracellular signals involved in anti-oxidation, anti-inflammation, and anti-apoptosis.

## Introduction

Ischemic cardiomyopathy is the leading cause of mortality and morbidity around the world (Murphy and Steenbergen, 2008). It is generally recognized that timely myocardial reperfusion using either thrombolytic therapy or primary percutaneous coronary intervention is effective in preserving life, limiting myocardial infarct size, preserving left-ventricular systolic function, and reducing the onset of heart failure; however, morbidity and mortality of patients with ischemic cardiomyopathy remain significant concerns due to the myocardial reperfusion-induced oxidative stress and subsequent inflammation and cell apoptosis (Frank et al., 2012; Heusch and Gersh, 2017). Cardioprotection against ischemia/reperfusion injury is urgently required to minimize infarct and subsequent heart failure (Frohlich et al., 2013). Although a number of ischemic and pharmacological cardioprotection strategies tested in experimental animals in the past three decades have been examined in the clinical setting to treat acute myocardial infarction and coronary artery bypass surgery, the results of many of the clinical studies have been disappointing, since no effective cardioprotective therapy exists in clinical practice (Hausenloy and Yellon, 2016; Heusch, 2017).

Great effort is needed to seek novel therapeutic strategies targeting myocardial oxidative stress and subsequent inflammation and apoptosis induced by ischemia/reperfusion injury (Heusch, 2015). Although our recent study have reported that the natural flavone acacetin and its water soluble prodrug, in addition to having anti-atrial fibrillation properties by selectively blocking multiple atrial potassium channels (Li et al., 2008; Wu et al., 2011, 2013; Liu et al., 2016a), provides remarkable cardioprotection against ischemia/reperfusion injury in *ex vivo* and *in vivo* rat models by increasing the anti-oxidants superoxide dismutase-2 (SOD2) and thioredoxin, and decreasing inflammation and apoptosis (Liu et al., 2016b); however, the underlying mechanism is not fully understood. In the present study, we investigate the potential signaling pathways of cardiomyocytes protection of acacetin against hypoxia/reoxygenation injury in two types of cells (neonatal rat cardiomyocytes and H9C2 cardiomyoblasts derived from embryonic BD1X rat heart tissue) using multiple biochemical approaches. Our results demonstrated that acacetin stimulated AMPK, which mediates activation of Nrf2 signal pathway in cardiomyocytes protection against hypoxia/reoxygenation injury via increasing the anti-oxidants heme oxygenase 1 (HO-1), SOD1 and SOD2, reducing ROS (reactive oxygen species) production, apoptotic and inflammation molecules thereby effectively inhibiting hypoxia/reoxygenation injury.

## Materials and Methods

### Reagents and Antibodies

Acacetin (5,7-dihydroxy-40-methoxyflavone) was synthesized in the laboratory as described previously in the United States patent (Li et al., 2010). Dulbecco’s modified Eagle’s medium (DMEM), fetal bovine serum (FBS), Lipofectamine 2000 reagents, and 2′7′-dichlorofluorescein diacetate (DCFH-DA) were purchased from Thermo Fisher Scientific (Waltham, MA, United States). The Annexin V/PI Apoptosis Detection Kit was obtained from Dojindo Molecular Technologies (Kumamoto, Japan). Accutase was from eBioscience (Santiago, CA, United States), Collagenase II was from Worthington Biochemical, Co. (Lakewood, NJ, United States). Small interfering RNA (siRNA) molecules targeting rat Nrf2 mRNA (sc-156128) was from Santa Cruz Biotechnology (Dallas, TX, United States), while siRNA molecules targeting rat AMPK and scrambled control siRNA were synthesized by RiboBio, Co., Ltd. (Guangzhou, Guangdong, China).

The anti-SOD2 (sc-133134), anti-Bcl-2 (sc-7382), anti-Bax (sc-493), anti-TLR-4 (sc-293072), anti-IL-10 (sc-365858), anti-AMPK (sc-25792), anti-pAMPK (sc-33524), anti-JNK (sc-571), anti-pJNK (sc-12882), and anti-β-actin (sc-130300) antibodies were purchased from Santa Cruz Biotechnology. The anti-Nrf2 (PB0327), anti-HO-1 (PB0212), anti-SOD1 (BA1401) and anti-IL-6 (PB0061) antibodies were from Wuhan Boster Biological Technology, Ltd. (Wuhan, Hubei, China), while the anti-cleaved caspase-3 antibody (9661S), anti-P38 (8690S), anti-pP38 (4511S), anti-ERK1/2 (4695S), anti-pERK1/2 (9101S), anti-Akt (9272S), anti-pAkt (4060S) antibodies were obtained from Cell Signaling Technology (Danvers, MA, United States).

### Primary Culture of Neonatal Rat Cardiomyocytes

The animal experiment protocol was approved by the Animal Care and Ethics Committee of Xiamen University. Sprague-Dawley (SD) rats (250–300 g, ♂ + ♀) were from Beijing Vital River Laboratory Animal Technology, Co. (Beijing, China) and mated naturally to produce offspring in Laboratory Animal Center of Xiamen University. The animals were cared for following the Guide for the Care and Use of Laboratory Animals published by the United States National Institutes of Health (NIH Publication No. 85-23, revised 1996). The hearts of 1- to 3-day-old neonatal rats were surgically removed under sterile conditions, washed three times with cold PBS, minced into ∼1 mm^3^ tissue chunks, and then digested with PBS containing collagenase II (0.1%) for 15–20 min at 37°C. The supernatants were transferred to a fresh sterile tube, the DMEM (containing 10% FBS) was added to stop digestion. The tissue chunks were then digested for 10 min with fresh Accutase (eBioscience) for two to three times (Weikert et al., 2003). After each digestion, cells were collected in DMEM and kept on ice. The isolated cells were filtered with a 100 μm cell strainer, seeded into culture dishes and incubated in DMEM for 1 h. Unattached cardiomyocytes were collected and seeded into 35 mm culture dishes, the cells were cultured in DMEM supplemented with 100 μM 5-bromo-2′-deoxyuridine (Sigma-Aldrich, St. Louis, MO, United States) for an additional 24 h. Cardiomyocytes were cultured for at least 3 days before hypoxia/reoxygenation exposure.

### Cell Culture and Hypoxia/Reoxygenation

Primary cultured neonatal rat cardiomyocytes and H9C2 cardiomyoblasts (ATCC, Manassas, VA, United States) were cultured at 37°C with 95% air and 5% CO_2_ in DMEM supplemented with 10% FBS, 100 μg/ml streptomycin, and 100 units/ml penicillin. When cells grew to 70–80% confluence, they were pretreated with acacetin (0.3, 1, and 3 μM) or vehicle (DMSO) for 4 h (a time point with better efficacy in cardiomyocytes protection). The cells were then exposed to an anaerobic medium (serum and glucose free) in a hypoxia incubator chamber (STEMCELL Technologies, Vancouver, BC, Canada) with an anoxic mixture gas (95% N_2_ and 5% CO_2_) for 18 h at 37°C followed by reoxygenation for 6 h with fresh culture medium (95% air and 5% CO_2_) to simulate ischemia/reperfusion injury in isolated hearts and/or in anesthetized rats (Liu et al., 2016b).

### Flow Cytometry Analysis

The flow cytometry analysis (Beckman Coulter, United States) was employed to determine cell viability, apoptosis, and intracellular ROS level as described previously (Wang Y. et al., 2016; Zhong and Tang, 2016). For cell viability and apoptosis analysis, primary neonatal rat cardiomyocytes and H9C2 cardiomyoblasts were cultured in the absence and presence of acacetin after hypoxia/reoxygenation exposure, and then determined by Annexin V-FITC Apoptosis Detection Kit (Dojindo Molecular Technologies, Inc., Rockville, MD, United States) according to the manufacturer’s instruction. Briefly, the collected cells were washed with PBS, then incubated at 4°C in a binding buffer (100 μl) with 5 μl Annexin V and 5 μl propidium iodide (PI) for 15 min at room temperature in the dark. Finally, the cells were analyzed by flow cytometry within 1 h.

For ROS production determination, neonatal rat cardiomyocytes, and H9C2 cardiomyoblasts were cultured in the absence and presence of acacetin. After hypoxia/reoxygenation exposure, the cells were incubated with DCFH-DA (10 μM) at 37°C for 30 min and intracellular ROS level was determined by a flow cytometer (Beckman Coulter, United States).

### Western Blot Analysis

The Western blots were employed using the procedure described previously (Wang Y. et al., 2016; Li et al., 2017) to determine the expression of Nrf2, HO-1, SOD1, SOD2, Bcl-2, Bax, cleaved caspase-3, TLR-4, IL-6, IL-10, P38, pP38, ERK1/2, pERK1/2, JNK, pJNK, Akt, pAkt, AMPK, pAMPK, and β-actin in neonatal rat cardiomyocytes and H9C2 cardiomyoblasts. Total proteins of the cell lysates were extracted by using protein extraction RIPA buffer with protease inhibitor. Protein concentration was estimated by using the BCA protein assay Kit (Solarbio, Beijing, China). Proteins samples were separated via SDS-PAGE and transferred to PVDF membranes (Bio-Rad, Hercules, CA, United States). The membranes were blocked and incubated with primary antibodies (1:1000) at 4°C overnight. After washing, membranes were incubated with secondary antibody (1:10000) at room temperature for 1 h. Blots were visualized with ECL^TM^ reagents (Advansta, Menlo Park, CA, United States), and the signals were captured with chemiluminescence detection system (FluoChem E, San Jose, CA, United States). The density of the band was analyzed with image analysis software.

### Silence of Nrf2 and AMPK

The experiments on silencing Nrf2 or AMPK were performed in H9C2 cardiomyoblasts as described previously (Wang Y. et al., 2016; Li et al., 2017). Briefly, when cells grew to 60–70% confluence, siRNA molecules were transfected into the cells using Lipofectamine 2000 reagent. Control siRNA was used to determine the efficiency of Nrf2 or AMPK specific siRNA transfection. 48 h after transfection, cells were pretreated with vehicle or acacetin (3 μM) and then exposed to 18 h hypoxia followed by 6 h reoxygenation. Afterward, the gene silencing efficacy and related proteins were determined with Western blot analysis.

### Myocardial Ischemia/Reperfusion Model in Anesthetized Rats

The myocardial ischemia/reperfusion model in anesthetized rats was established as described previously (Liu et al., 2016b) to confirm the AMPK involvement of acacetin in cardiomyocytes protection against hypoxia/reoxygenation insult through Nrf2 using the AMPK inhibitor Compound C (Novikova et al., 2015). Adult male SD rats (250–300 g) were anesthetized with pentobarbital (50 mg/kg i.p.), supplemented during the experiment when needed. The animals were incubated and ventilated with room air. Body temperature was maintained at 37°C with a temperature control system. Regional ischemia was achieved by ligating left anterior descending (LAD) artery using a 5-0 silk suture with a section of silica gel tubing. Myocardial ischemia was confirmed by regional cyanosis and ST-segment elevation. After 10-min stabilization, 10 mg/kg acacetin prodrug, which is effectively converted to acacetin (Liu et al., 2016b) or equivolume vehicle (0.9% saline) was subcutaneously administered before LAD artery was ligated for 30 min, followed by a 120-min reperfusion. Compound C (1 mg/kg) was intraperitoneally administered after the animal was anesthetized. The cardiac tissues of ischemic zone were collected for Nrf2 protein expression analysis.

### Statistical Analysis

Data analysis was performed with GraphPad Prism 5.0 (GraphPad Software, Inc., San Diego, CA, United States). Results are presented as mean ± SEM. One-way ANOVA followed by Bonferroni-test was used for comparisons of multiple groups. A value of *P* < 0.05 was considered as statistically significant.

## Results

### Effects of Acacetin on Cell Viability and Apoptosis in Cells Subjected to Hypoxia/Reoxygenation Insult

**FIGURE 1:**
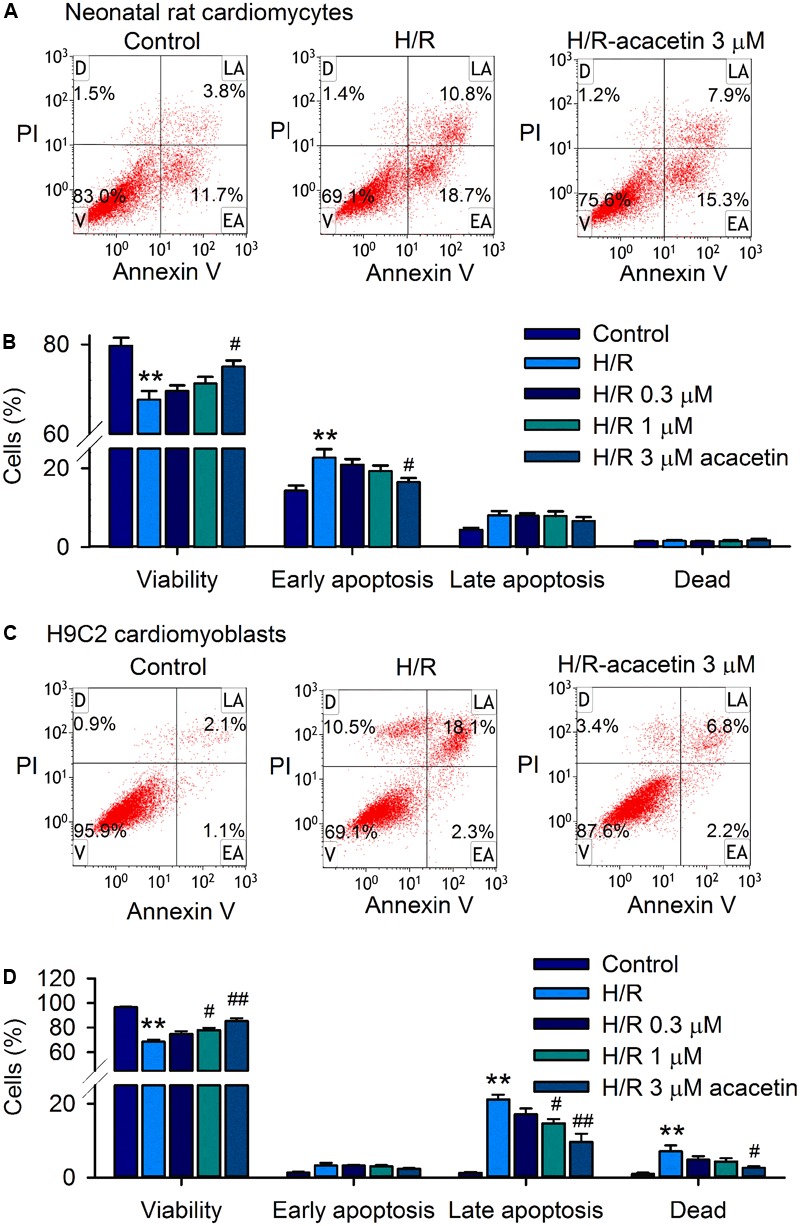
Effects of acacetin on cell viability and apoptosis in neonatal rat cardiomyocytes and H9C2 cardiomyoblasts subjected to hypoxia/reoxygenation. **(A)** Flow cytometry graphs showing cell viability and apoptosis populations in neonatal rat cardiomyocytes without (control) or with hypoxia/reoxygenation (H/R) exposure in the absence or presence of 3 μM acacetin. Cells were treated with FITC-labeled Annexin V and PI staining [viability (V); dead cells (D); late apoptosis (LA); early apoptosis (EA)]. **(B)** Mean percent values of cell viability, early apoptosis, late apoptosis, and dead cells in neonatal rat cardiomyocytes without or with hypoxia/reoxygenation (H/R) in the absence or presence of 0.3, 1, or 3 μM acacetin. **(C)** Flow cytometry graphs of H9C2 cardiomyoblasts with the treatment used in **(A)**. **(D)** Mean percent values of cell viability, early apoptosis, late apoptosis, and dead cells in H9C2 cardiomyoblasts with the treatment used in **(A)**. Data were expressed as mean ± SEM and analyzed by one-way ANOVA followed by Bonferroni-test (*n* = 5 individual experiments, ^∗∗^*P* < 0.01 vs. control; ^#^*P* < 0.05, ^##^*P* < 0.01 vs. H/R alone).

Acacetin had no effect on cell viability in H9C2 cardiomyoblasts with 24 h incubation (Supplementary Figure S1). However, it antagonized the reduction of viability and the increase of apoptosis induced by hypoxia/reoxygenation insult in primary neonatal rat cardiomyocytes and H9C2 cardiomyoblasts subjected to 18 h hypoxia followed by 6 h reoxygenation. **Figure 1A** shows the flow cytometry graphs of neonatal rat cardiomyocytes without or with hypoxia/reoxygenation exposure in the absence or presence of 3 μM acacetin. Cell viability was 83% with 11.7% early apoptosis under control conditions. Hypoxia/reoxygenation insult decreased the viability to 69.1% and increased early apoptosis to 18.7%. Acacetin at 3 μM partially reversed the reduced viability to 75.6% and the increased early apoptosis to 15.3%. **Figure 1B** illustrates the mean percent values of cell viability and apoptosis. Acacetin at 0.3–3 μM increased cell viability and decreased early apoptosis. A statistically significant effect was observed at 3 μM (*P* < 0.05 vs. hypoxia/reoxygenation).

In H9C2 cardiomyoblasts, hypoxia/reoxygenation insult decreased cell viability and increased late apoptosis and dead cells, which were partially antagonized by pre-incubation of 3 μM acacetin (**Figure 1C**). Statistical analysis (**Figure 1D**) shows that cell viability was decreased from 96.5 ± 0.6% of control to 68.5 ± 1.8% (*n* = 5, *P* < 0.01 vs. control), and the late apoptotic cells were increased from 1.3 ± 0.2% of control to 21.2 ± 1.3% (*P* < 0.01 vs. control), and the dead cells from 0.9 ± 0.4% of control to 7.1 ± 1.6% (*P* < 0.01 vs. control) in cells with hypoxia/reoxygenation exposure. Acacetin at 0.3, 1, and 3 μM antagonized the reduction of cell viability and the increased apoptotic cells and dead cells. Significant effect was observed at 1 and 3 μM for cell viability and late apoptotic cells (*n* = 5, *P* < 0.05 or *P* < 0.01 vs. hypoxia/reoxygenation alone) and at 3 μM for dead cells (*P* < 0.05 vs. hypoxia/reoxygenation alone).

These results suggest that hypoxia/reoxygenation insult reduces cell viability in both primary neonatal rat cardiomyocytes and H9C2 cardiomyoblasts. Early apoptosis was seen in neonatal rat cardiomyocytes, while late apoptosis and dead cells were observed in H9C2 cardiomyoblasts. Acacetin at 3 μM significantly antagonized the apoptosis induced by hypoxia/reoxygenation insult.

### Effects of Acacetin on Anti-apoptotic and Pro-apoptotic Proteins

**FIGURE 2:**
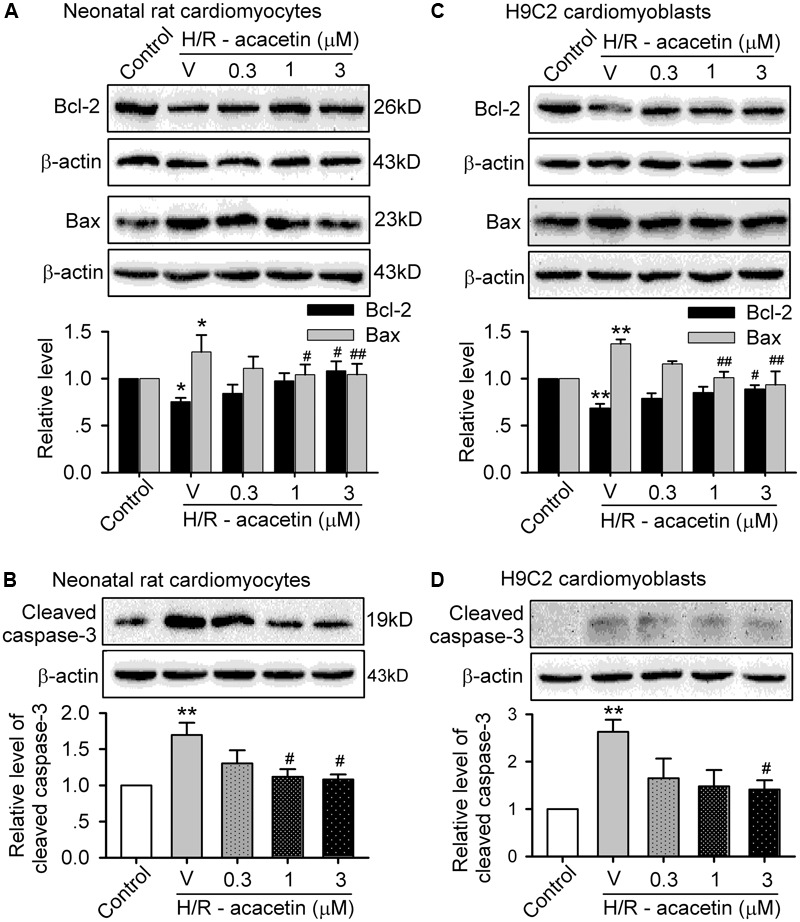
Effects of acacetin on apoptosis-related proteins in cells with hypoxia/reoxygenation exposure. **(A)** Western blots and mean relative level of Bcl-2 and Bax in neonatal rat cardiomyocytes without (control) or with hypoxia/reoxygenation (H/R) exposure in the absence (V, vehicle) or presence of 0.3, 1, or 3 μM acacetin. **(B)** Western blots and mean relative level of cleaved caspase-3 in neonatal rat cardiomyocytes with the treatment as in **(A)**. **(C)** Western blots and mean relative level of Bcl-2 and Bax in H9C2 cardiomyoblasts with the treatment used in **(A)**. **(D)** Western blots and mean relative level of cleaved caspase-3 in H9C2 cardiomyoblasts with the treatment used in **(A)**. Data were expressed as mean ± SEM and analyzed by one-way ANOVA followed by the Bonferroni-test (*n* = 5 individual experiments, ^∗^*P* < 0.05, ^∗∗^*P* < 0.01 vs. control group; ^#^*P* < 0.05, ^##^*P* < 0.01 vs. hypoxia/reoxygenation alone).

The reduction of cell viability mainly results from apoptosis in both primary neonatal rat cardiomyocytes and H9C2 cardiomyoblasts when subjected to hypoxia/reoxygenation insult. The proteins related to anti-apoptosis and pro-apoptosis (Liu et al., 2016b) were therefore determined by Western blotting analysis. **Figure 2** displays the expression levels of the anti-apoptotic protein Bcl-2 and the pro-apoptotic proteins Bax and cleaved caspase-3 in cells without or with hypoxia/reoxygenation exposure in the absence and presence of 0.3, 1, and 3 μM acacetin. In primary neonatal rat cardiomyocytes (**Figures 2A,B**), hypoxia/reoxygenation insult, as in ischemia/reperfusion insult (Liu et al., 2016b), down-regulated Bcl-2 while up-regulating Bax and cleaved caspase-3. Acacetin rescued the down-regulated Bcl-2 and decreased the up-regulated Bax and cleaved caspase-3. Similar results were observed in H9C2 cardiomyoblasts (**Figures 2C,D**), in which hypoxia/reoxygenation decreased Bcl-2 and increased Bax and cleaved caspase-3. Acacetin treatment antagonized the alterations by hypoxia/reoxygenation insult. These results confirmed that acacetin may antagonize the apoptosis induced by hypoxia/reoxygenation insult, as previously observed in rat hearts subjected to ischemia/reperfusion insult (Liu et al., 2016b).

### Effects of Acacetin on Cytokines Related to Inflammation

**FIGURE 3:**
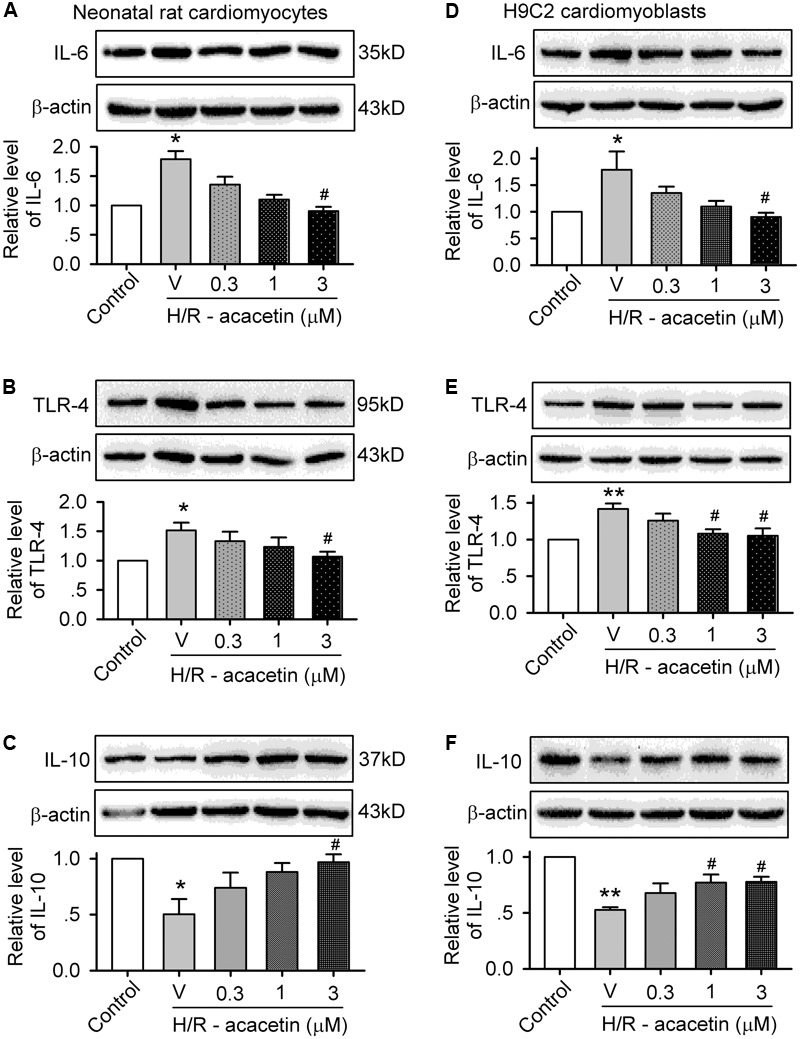
Effects of acacetin on inflammation-related cytokines in cells with hypoxia/reoxygenation exposure. Western blots and mean relative level of IL-6 **(A)**, TLR-4 **(B)**, IL-10 **(C)** in neonatal rat cardiomyocytes without (control) or with hypoxia/reoxygenation (H/R) exposure in the absence (V, vehicle) or presence of 0.3, 1, or 3 μM acacetin. Western blots and mean relative level of IL-6 **(D)**, TLR-4 **(E)**, IL-10 **(F)** in H9C2 cardiomyoblasts with the treatment used in **(A–C)**. Data were expressed as mean ± SEM and analyzed by one-way ANOVA followed by the Bonferroni-test (*n* = 5 individual experiments, ^∗^*P* < 0.05, ^∗∗^*P* < 0.01 vs. control; ^#^*P* < 0.05 vs. hypoxia/reoxygenation alone).

To investigate whether anti-inflammation is involved in the anti-apoptotic effect of acacetin in cells subjected to hypoxia/reoxygenation insult, the expression levels of the pro-inflammatory factors IL-6 and TLR-4 and the anti-inflammatory factor IL-10 were determined in primary neonatal rat cardiomyocytes and H9C2 cardiomyoblasts without or with hypoxia/reoxygenation exposure in the absence and presence of 0.3, 1, and 3 μM acacetin (**Figure 3**). In primary neonatal rat cardiomyocytes (**Figures 3A–C**), hypoxia/reoxygenation caused a significant increase of IL-6 and TLR-4 and decrease of IL-10, and these effects were reversed by acacetin. Similar results were seen in H9C2 cardiomyoblasts (**Figures 3D–F**), in which the increased IL-6 and TLR-4 and the decreased IL-10 were significantly reversed by acacetin. These results suggest that acacetin-induced anti-inflammatory effect may participate in the anti-apoptosis in both primary neonatal rat cardiomyocytes and H9C2 cardiomyoblasts subjected to hypoxia/reoxygenation insult.

### Effect of Acacetin on Hypoxia/Reoxygenation-Induced ROS Production

**FIGURE 4:**
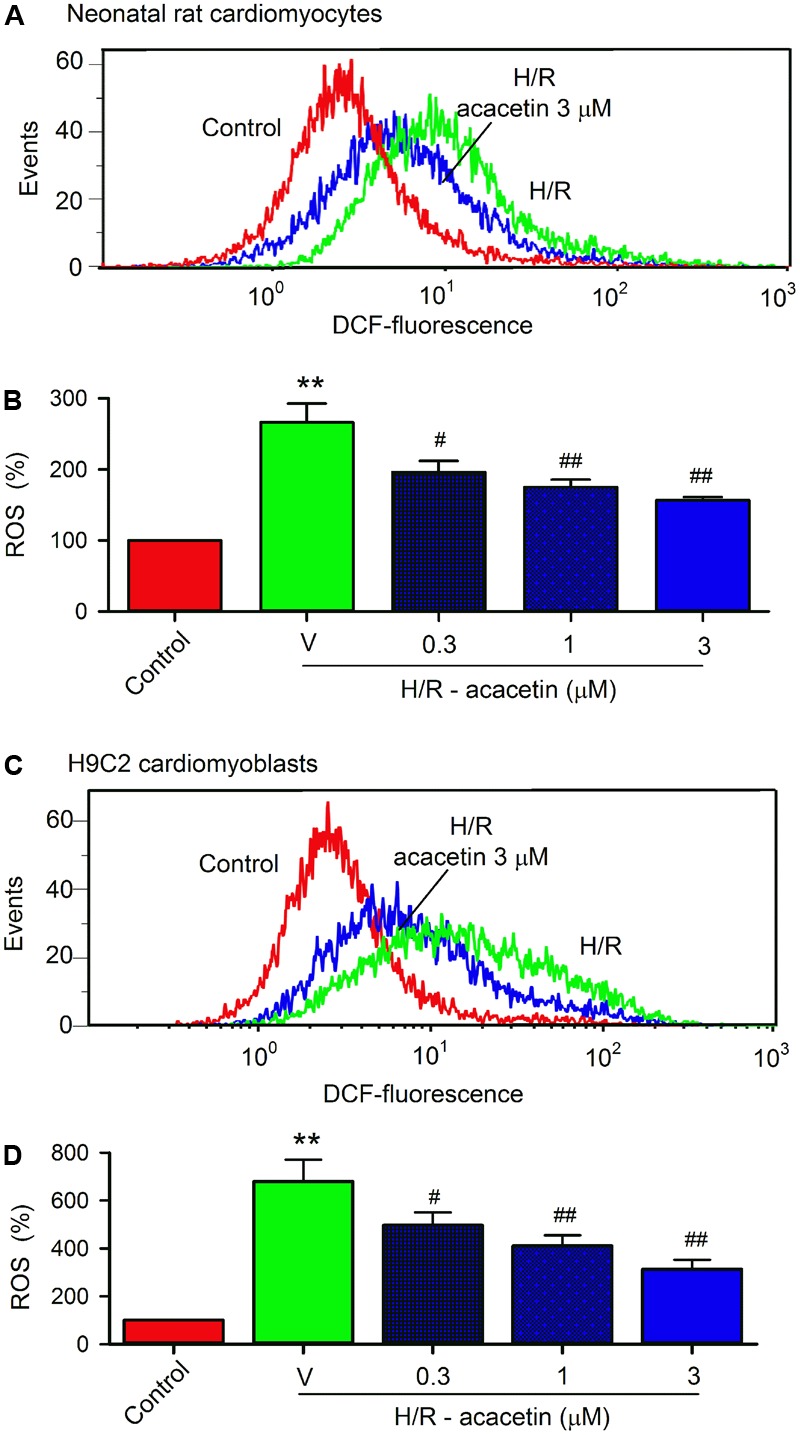
Effect of acacetin on intracellular ROS production in cells with hypoxia/reoxygenation exposure. **(A)** Flow cytometry graphs showing ROS production in neonatal rat cardiomyocytes without (control) or with hypoxia/reoxygenation (H/R) exposure in the absence or presence of 3 μM acacetin. **(B)** Mean percent values of ROS production in neonatal rat cardiomyocytes without (control) or with hypoxia/reoxygenation (H/R) exposure in the absence (V, vehicle) or presence of 0.3, 1, or 3 μM acacetin. **(C)** Flow cytometry graphs showing ROS production in H9C2 cardiomyoblasts with the treatment used in **(A)**. **(D)** Mean percent values of ROS production in H9C2 cardiomyoblasts with the treatment used in **(B)**. Data were expressed as mean ± SEM and analyzed by one-way ANOVA followed by the Bonferroni-test (*n* = 5 individual experiments, ^∗∗^*P* < 0.01 vs. control; ^#^*P* < 0.05 and ^##^*P* < 0.01 compared with the hypoxia/reoxygenation alone).

It is generally believed that ROS play a crucial role in mediating ischemia/reperfusion or hypoxia/reoxygenation injury by initiating inflammation and apoptosis. To study whether the protective effect of acacetin is related to inhibiting intracellular ROS production, the cells were loaded with dichlorofluorescin (Kooy et al., 1997) and ROS level was determined by flow cytometry. **Figure 4** shows the ROS production in primary neonatal rat cardiomyocytes and H9C2 cardiomyoblasts without or with hypoxia/reoxygenation exposure in the absence and presence of acacetin. ROS production was greatly increased in cells subjected to hypoxia/reoxygenation insult, and acacetin significantly decreased the ROS production. In primary neonatal rat cardiomyocytes, hypoxia/reoxygenation insult increased intracellular ROS level to 266.3 ± 26.3% of control (**Figure 4B**), which was decreased by 3 μM acacetin to 156.4 ± 4.5% of control (*n* = 5, *P <* 0.01 vs. hypoxia/reoxygenation alone). Similar results were observed in H9C2 cardiomyoblasts (**Figure 4C**), in which acacetin (3 μM) reduced the ROS level from 676.3 ± 90.8% of control in cells with hypoxia/reoxygenation insult to 313.1 ± 38.9% (*n* = 5, *P <* 0.01 vs. hypoxia/reoxygenation alone) (**Figure 4D**). These results indicate that acacetin significantly inhibits ROS production in both primary neonatal rat cardiomyocytes and H9C2 cardiomyoblasts subjected to hypoxia/reoxygenation insult.

### Effects of Acacetin on Nrf2/HO-1 Antioxidative Pathway

It is well-recognized that intracellular ROS level is dependent on Nrf2 to regulate endogenous antioxidants, e.g., HO-1, SOD1, and SOD2, etc. (Gold et al., 2012; Ma, 2013). We therefore determined whether acacetin would increase the expression of the regulator Nrf2, and also the antioxidant HO-1, SOD1, or SOD2 expression in primary neonatal rat cardiomyocytes and H9C2 cardiomyoblasts. Acacetin at 0.3, 1, and 3 μM increased Nrf2 and HO-1, and significant effect was observed at 1 and 3 μM. Acacetin only slightly increased SOD1, and its increase of SOD2 was significant at 3 μM (Supplementary Figure S2). These results suggest that acacetin may stimulate Nrf2/HO-1 antioxidative pathway.

**FIGURE 5:**
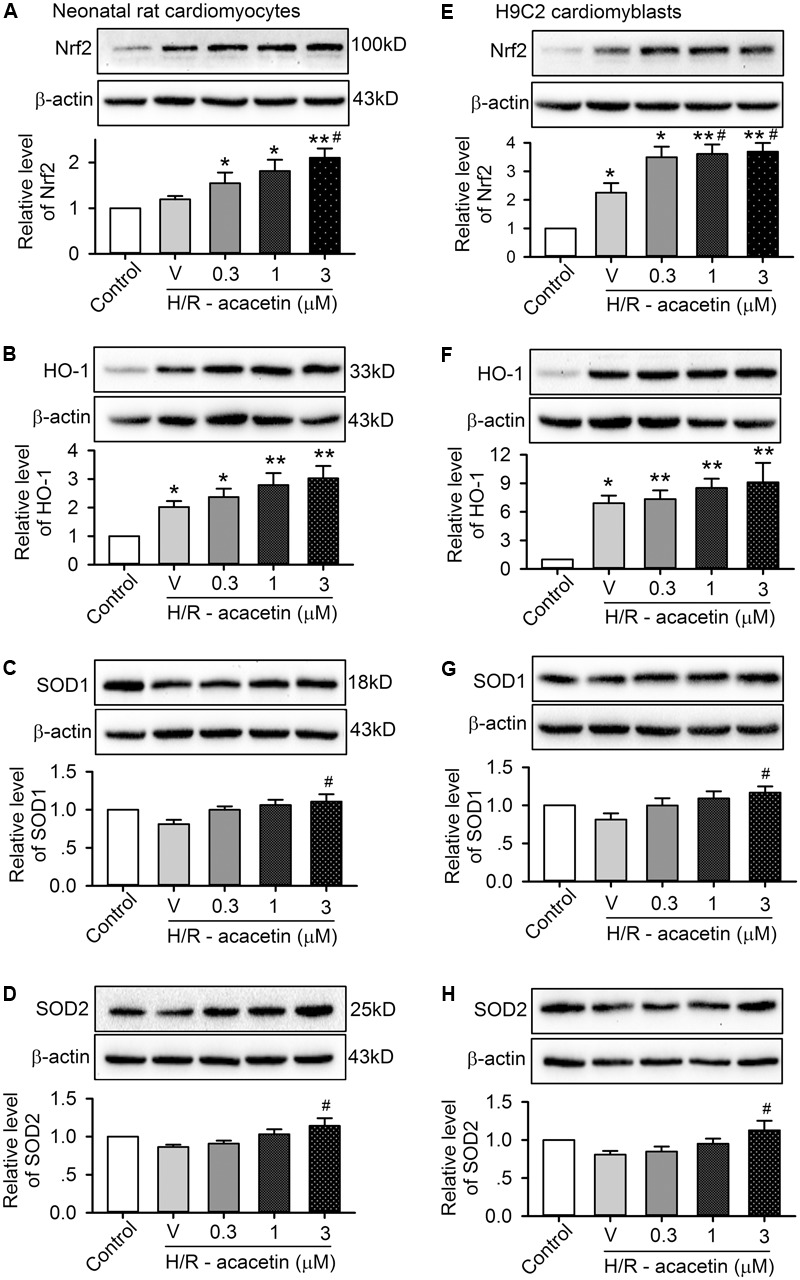
Effects of acacetin on antioxidant-related proteins in cells with hypoxia/reoxygenation (H/R) exposure. Western blots and mean relative levels of Nrf2 **(A)**, HO-1 **(B)**, SOD1 **(C)**, SOD2 **(D)** in neonatal rat cardiomyocytes without (control) or with hypoxia/reoxygenation exposure in the absence (V, vehicle) or presence of 0.3, 1, or 3 μM acacetin. Western blots and mean relative levels of Nrf2 **(E)**, HO-1 **(F)**, SOD1 **(G)**, SOD2 **(H)** in H9C2 cardiomyoblasts without (control) or with hypoxia/reoxygenation exposure in the absence or presence of 0.3, 1, or 3 μM acacetin. Data were expressed as mean ± SEM and analyzed by one-way ANOVA followed by Bonferroni-test (*n* = 5 individual experiments, ^∗^*P* < 0.05, ^∗∗^*P* < 0.01 vs. control; ^#^*P* < 0.05 vs. hypoxia/reoxygenation alone).

The effect of acacetin on Nrf2/HO-1 antioxidative pathway was then determined in primary neonatal rat cardiomyocytes and H9C2 cardiomyoblasts subjected to hypoxia/reoxygenation insult (**Figure 5**). Hypoxia/reoxygenation insult stimulated an increase in expression of Nrf2 and HO-1 in both types of cells. Acacetin at 0.3–3 μM induced an additional increase in a concentration-dependent manner. However, hypoxia/reoxygenation insult slightly decreased SOD1 and SOD2, and the reduction was reversed in cells treated with 3 μM acacetin. These results suggest that upregulation of Nrf2, HO-1, SOD1, and SOD2 may be involved in acacetin protection of cardiomyocytes against hypoxia/reoxygenation in primary neonatal rat cardiomyocytes and H9C2 cardiomyoblasts.

### Nrf2 and Cardiomyocytes Protection of Acacetin Against Hypoxia/Reoxygenation Injury

Nrf2 regulates the expression of antioxidant pathway proteins that protect against oxidative damage triggered by injury (Gold et al., 2012; Ma, 2013). To investigate whether Nrf2 is a crucial player in mediating acacetin protection of cardiomyocytes against hypoxia/reoxygenation insult, siRNA molecules targeting Nrf2 were transfected into H9C2 cardiomyoblasts. Afterward, the H9C2 cardiomyoblasts were subjected to hypoxia/reoxygenation insult to determine the effects of acacetin on cell viability, apoptosis, ROS production, apoptosis- and inflammation-related proteins.

**FIGURE 6:**
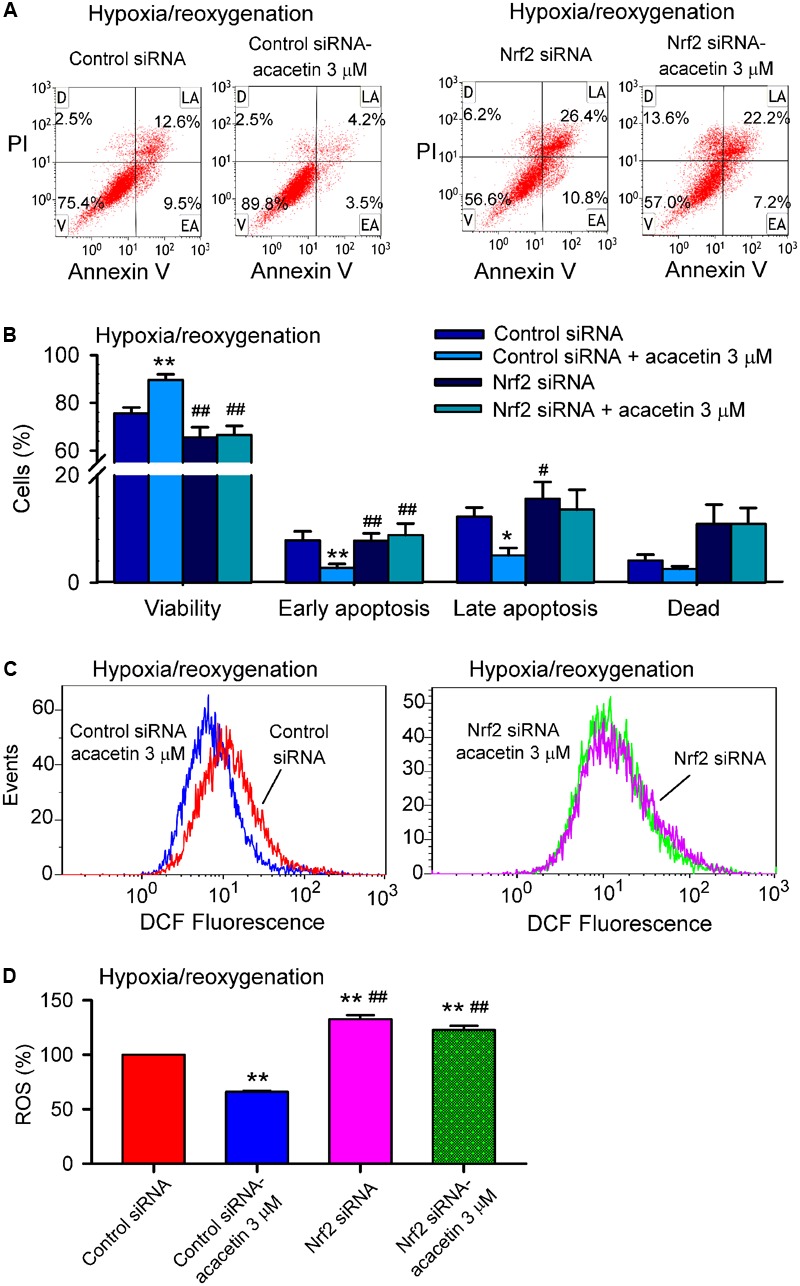
Silencing Nrf2 abolished cardiomyocytes protection of acacetin against hypoxia/reoxygenation insult. **(A)** Flow cytometry graphs showing cell viability, early apoptosis, late apoptosis, and dead cells in H9C2 cardiomyoblasts transfected with control siRNA or Nrf2 siRNA for 48 h, then subjected to hypoxia/reoxygenation in the absence (vehicle control) or presence of 3 μM acacetin. **(B)** Mean percent values of cell viability, early apoptosis, late apoptosis, and dead cells in H9C2 cardiomyoblasts transfected with control siRNA or Nrf2 siRNA for 48 h, then subjected to hypoxia/reoxygenation in the absence (vehicle control) or presence of 3 μM acacetin. **(C)** Flow cytometry graphs showing ROS production in H9C2 cardiomyoblasts with the treatment used in **(A)**. **(D)** Mean percent values of ROS production in H9C2 cardiomyoblasts. Data were expressed as mean ± SEM and analyzed by one-way ANOVA followed by Bonferroni-test (*n* = 5 individual experiments, ^∗^*P* < 0.05, ^∗∗^*P* < 0.01 vs. control siRNA; ^#^*P* < 0.05, ^##^*P* < 0.01 vs. control siRNA with acacetin).

Hypoxia/reoxygenation insult resulted in the reduced cell viability and increased apoptotic cell population, acacetin (3 μM) antagonized these effects in cells transfected with control siRNA, but not in cells transfected with Nrf2 siRNA (**Figures 6A,B**). In addition, intracellular ROS production induced by hypoxia/reoxygenation insult was reduced by acacetin in cells transfected with control siRNA (**Figures 6C,D**); however, in cells transfected with Nrf2 siRNA, hypoxia/reoxygenation-induced a greater increase of ROS level (*n* = 5, *P* < 0.01 vs. control siRNA), which cannot be decreased by acacetin. These results suggest that cardiomyocytes protection of acacetin against hypoxia/reoxygenation is related to Nrf2 activation.

**FIGURE 7:**
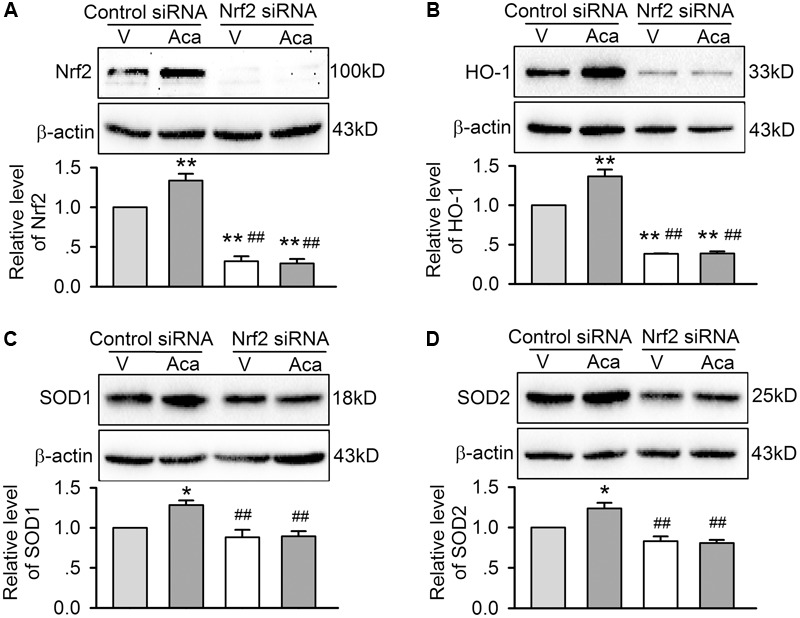
Effects of silencing Nrf2 on antioxidant proteins in cells with hypoxia/reoxygenation insult. **(A)** Western blots and relative levels of Nrf2 in H9C2 cardiomyoblasts transfected with control siRNA or Nrf2 siRNA and subjected to hypoxia/reoxygenation insult in the absence (V, vehicle) or presence of 3 μM acacetin (Aca). **(B)** Western blots and relative levels of HO-1 in H9C2 cardiomyoblasts with the treatment used in **(A)**. **(C)** Western blots and relative levels of SOD1 in H9C2 cardiomyoblasts with the treatment used in **(A)**. **(D)** Western blots and relative levels of SOD2 in H9C2 cardiomyoblasts with the treatment used in **(A)**. Data were expressed as mean ± SEM and analyzed by one-way ANOVA followed by Bonferroni-test (*n* = 5 individual experiments, ^∗^*P* < 0.05, ^∗∗^*P* < 0.01 vs. vehicle of control siRNA; ^##^*P* < 0.01 vs. control siRNA with acacetin).

Nrf2 regulates the expression of downstream proteins (e.g., HO-1, SOD1, and SOD2, etc.). Therefore, these proteins related to apoptosis and inflammation were determined in H9C2 cardiomyoblasts transfected with control siRNA or Nrf2 siRNA molecules and subjected to hypoxia/reoxygenation insult (**Figure 7**). Nrf2 was significantly increased by 3 μM acacetin in cells transfected with control siRNA; silencing Nrf2 with Nrf2 siRNA removes acacetin effect and suggests that the target molecule of acacetin may be Nrf2 (**Figure 7A**). Interestingly, HO-1 expression was significantly decreased in cells transfected with Nrf2 siRNA. In cells transfected with control siRNA, HO-1 was enhanced by acacetin (**Figure 7B**). This further indicates that acacetin-induced HO-1 increase is dependent on Nrf2 activation.

On the other hand, the expression of SOD1 and SOD2 was slightly reduced in cells transfected with Nrf2 siRNA (**Figures 7C,D**), suggesting they are also regulated by Nrf2. SOD1 and SOD2 were increased by acacetin in cells transfected with control siRNA, but not in cells transfected with Nrf2 siRNA, which supports the notion that SOD1 and SOD2 are also regulated by acacetin via activating Nrf2.

**FIGURE 8:**
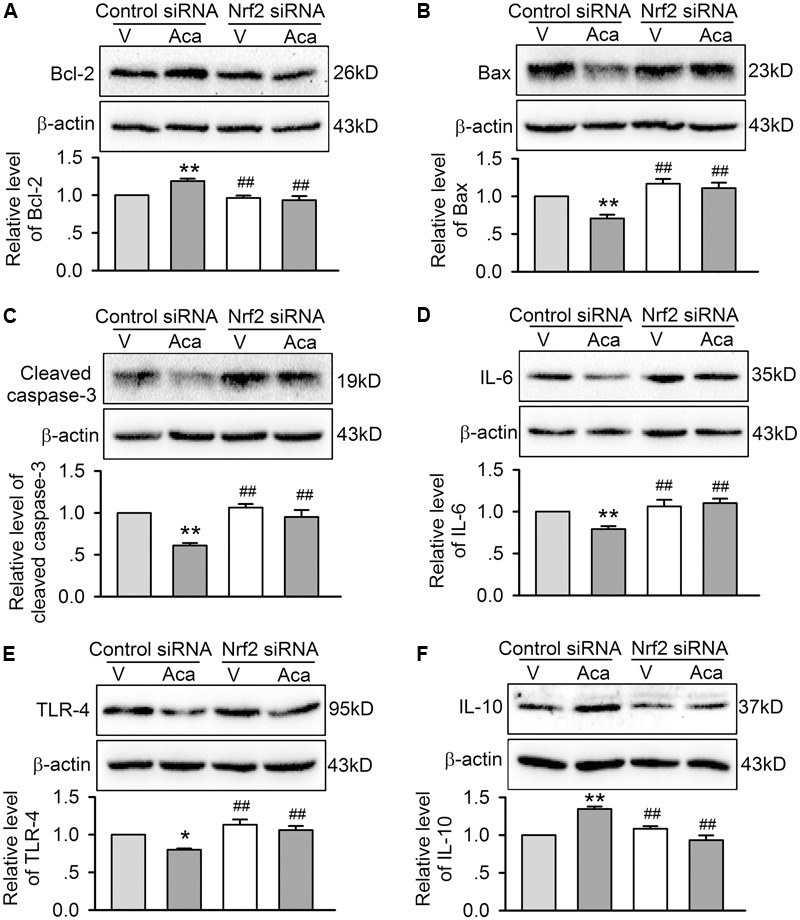
Effects of silencing Nrf2 on apoptosis- and inflammation-related proteins in cells with hypoxia/reoxygenation insult. Western blots and relative levels of Bcl-2 **(A)**, Bax **(B)**, and cleaved caspase-1 **(C)** in H9C2 cardiomyoblasts transfected with control siRNA or Nrf2 siRNA and subjected to hypoxia/reoxygenation insult in the absence (V, vehicle) or presence of 3 μM acacetin (Aca). Western blots and relative levels of IL-6 **(D)**, TRL-4 **(E)**, and IL-10 **(F)** in H9C2 cardiomyoblasts with the treatment used in **(A–C)**. Data were expressed as mean ± SEM and analyzed by one-way ANOVA followed by Bonferroni-test (*n* = 5 individual experiments, ^∗^*P* < 0.05, ^∗∗^*P* < 0.01 vs. vehicle of control siRNA; ^##^*P* < 0.01 vs. control siRNA with acacetin).

In addition, the beneficial effects of acacetin for reducing Bax, cleaved caspase-3, TLR-4 and IL-6, and upregulating Bcl-2 and IL-10 in cells subjected to hypoxia/reoxygenation insult disappeared in cells with silenced Nrf2 (**Figures 8A–F**), indicating that anti-apoptosis and anti-inflammation effects of acacetin are secondary to its activation of Nrf2 and subsequent anti-oxidation and reduction of intracellular ROS level.

### AMPK-Mediating the Activation of Nrf2 Signal Pathway by Acacetin

Recent studies demonstrated that several molecular kinases including P38 (Li et al., 2016), Akt (Hu et al., 2016), JNK and ERK1/2 (Lee et al., 2014), and AMPK (Park et al., 2016), have been implicated in mediating the activation of Nrf2 by different compounds. To determine which signal molecule mediates Nrf2 activation by acacetin, the effects of acacetin on the phosphorylation of these molecule kinases were determined in H9C2 cardiomyoblasts. However, acacetin at 0.3, 1, and 3 μM had no effect on pP38, pJNK, pERK1/2, and pAkt (Supplementary Figure S3).

**FIGURE 9:**
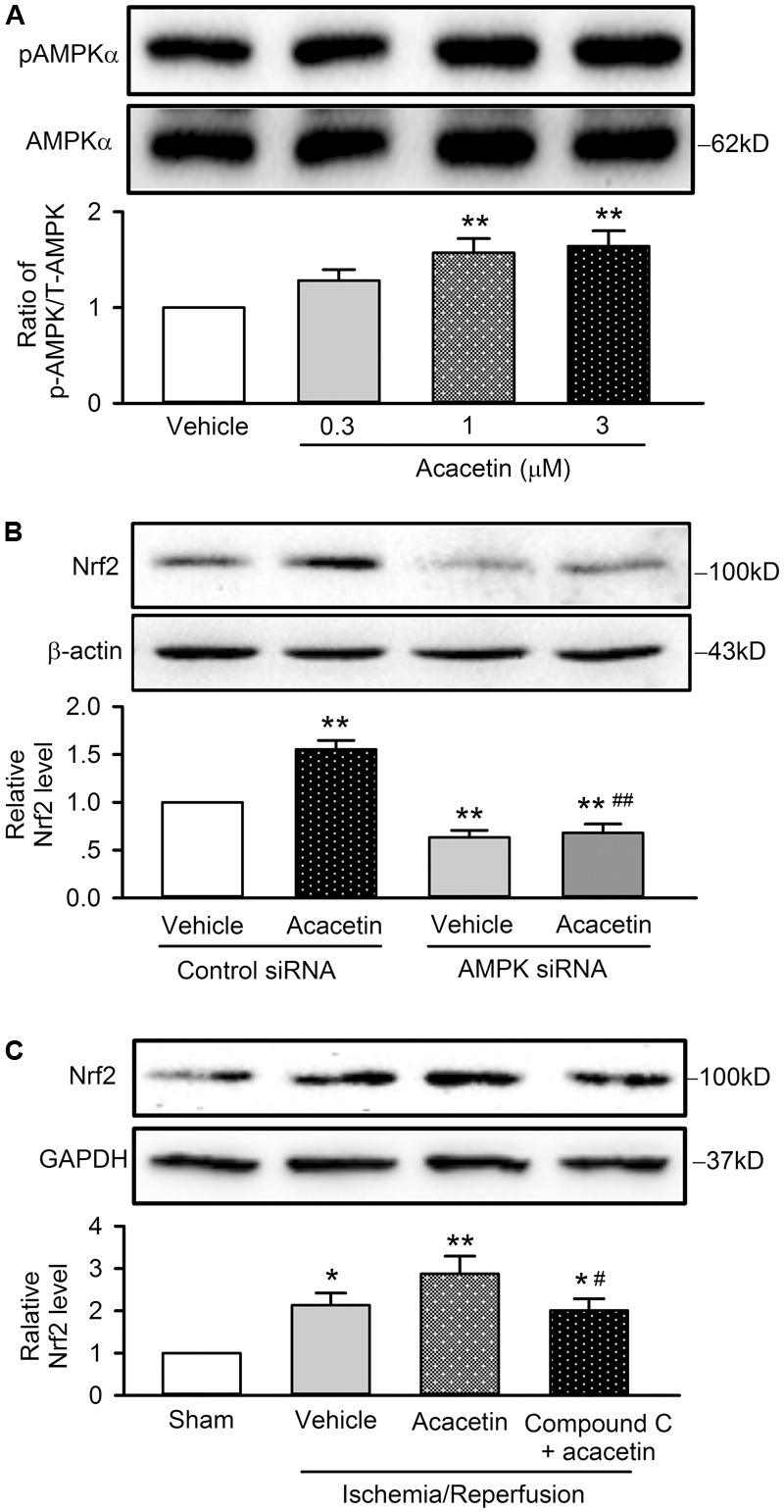
Nrf2 activation induced by acacetin is mediated by AMPK. **(A)** Western blots and relative level of pAMPK in H9C2 cardiomyoblasts treated by 0.3, 1, or 3 μM acacetin. **(B)** Western blots and relative level of Nrf2 in H9C2 cardiomyoblasts transfected with control siRNA or AMPK siRNA in the absence or presence of 3 μM acacetin. **(C)** Western blots and relative level of Nrf2 in left ventricular tissues of rats subjected to ischemia/reperfusion injury with treatment of acacetin or Compound C plus acacetin. Data were expressed as mean ± SEM and analyzed by one-way ANOVA followed by Bonferroni-test (*n* = 5 individual experiments, ^∗^*P* < 0.05 vs. sham, ^∗∗^*P* < 0.01 vs. control or vehicle of control siRNA or sham; ^#^*P* < 0.05 vs. acacetin; ^##^*P* < 0.01 vs. control siRNA with acacetin).

Interestingly, acacetin increased pAMPKα in a concentration dependent manner (**Figure 9A**), suggesting that AMPK may be an activator of Nrf2 signal pathway. To confirm whether acacetin mediates Nrf2 activation through AMPK, the effect of acacetin on Nrf2 expression was determined in cells transfected with siRNA targeting AMPKα. Acacetin (3 μM) increased Nrf2 expression in cells transfected with scramble control siRNA. Interestingly, Nrf2 expression was significantly reduced in cells transfected with siAMPKα and acacetin no longer increased Nrf2 in these cells (**Figure 9B**). The results indicate that acacetin mediates the activation of Nrf2 through AMPK.

The AMPK involvement of acacetin in cardiomyocytes protection against hypoxia/reoxygenation insult through Nrf2 was further confirmed in anesthetized rats subjected to ischemia/reperfusion injury, in which the animal was pretreated with the AMPK inhibitor Compound C (1 mg/kg). **Figure 9C** shows that cardiac ischemia/reperfusion increased Nrf2 expression (*n* = 5, *P* < 0.05 vs. sham), acacetin induced an additional increase of Nrf2 level (*P* < 0.01 vs. sham); however, the increase of Nrf2 by acacetin was not observed in rats treated with the AMPK inhibitor Compound C. The results from the *in vivo* animal support the notion that cardioprotection of acacetin against ischemia/reperfusion injury is mediated by AMPK/Nrf2 signal pathway.

## Discussion

**FIGURE 10:**
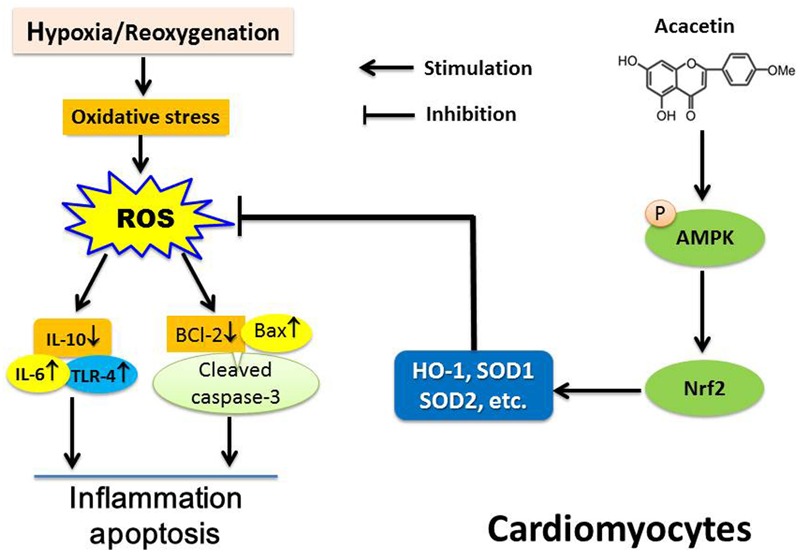
Schematic illustration showing the main cascade of molecules in cardiomyocytes subjected to ischemia/reperfusion insult and the mechanism of acacetin effects. Cardiomyocytes suffering from hypoxia/reoxygenation induces oxidative stress and triggers ROS production that induces inflammation and apoptosis via releasing the pro-inflammatory cytokines TLR-4 and IL-6 which increase the apoptotic proteins Bax and cleaved caspase-3, and decrease anti-inflammatory factor IL-10 and anti-apoptotic protein Bcl-2. Acacetin activates Nrf2 by stimulating AMPK, and increasing HO-1, SOD1, and SOD2, thereby conferring protection to cardiomyocytes against hypoxia/reoxygenation insult by reducing ROS production and inhibiting inflammation and apoptosis.

In the present study, we have demonstrated that the natural flavone acacetin significantly antagonizes hypoxia/reoxygenation-induced reduction of cell viability and increase of apoptosis in primary neonatal rat cardiomyocytes and H9C2 cardiomyoblasts. The cardioprotective effect of acacetin against hypoxia/reoxygenation insult is related to inhibiting ROS production. Acacetin activates pAMPKα which increases the master anti-oxidant regulator Nrf-2, thereby exerting anti-inflammatory and anti-apoptotic effects via increasing the anti-inflammatory cytokine IL-10 and the anti-apoptotic kinase Bcl-2 and decreasing the pro-inflammatory cytokines TLR-4 and IL-6 and the pro-apoptotic kinases Bax and cleaved caspase-3 (**Figure 10**).

Acacetin is a natural flavone (5,7-dihydroxy-4′-methoxyflavone) broadly distributed in plant pigments, and is responsible for many of the colors in nature (Cody, 1988). In addition to its anti-atrial fibrillation properties (Li et al., 2008; Liu et al., 2016a) by selectively blocking atrial potassium channels including I_Kur_ (ultra-rapidly activating delayed rectifier potassium current) or Kv1.5, I_K.ACh_ (acetylcholine-activated potassium current), I_to_ (transient outward potassium current) or Kv4.3 (Li et al., 2008; Wu et al., 2011, 2013) and SK_Ca_ current (small conductance Ca^2+^-activated potassium current) (Chen et al., 2017), acacetin has beneficial effects on lipopolysaccharide-induced neuroinflammation (Ha et al., 2012), inflammation-associated tumorigenesis (Pan et al., 2006), and acute lung injury (Wu et al., 2017) in mouse models. Our recent study (Liu et al., 2016b) demonstrated that acacetin and its prodrug are cardioprotective against ischemia/reperfusion injury in *ex vivo* and/or *in vivo* rat heart models, in which acacetin administration shows significant reduction of ventricular fibrillation and myocardial infarct size induced by ischemia/reperfusion insult via inhibiting inflammation and apoptosis (Liu et al., 2016b). However, the exact signaling pathway through which acacetin confers cardioprotection was not clarified in the previous report. The present study utilized a hypoxia/reoxygenation cell model and analyzed potential molecules involved in cardioprotective effects of acacetin.

It has been well-documented that ROS and redox signaling are involved in myocardial ischemia-reperfusion injury and cardioprotection (Cadenas, 2018). ROS are chemically reactive oxygen species including peroxides, superoxide, hydroxyl radical, and singlet oxygen (Hayyan et al., 2016), which are generated as a natural byproduct of normal oxygen metabolism and have important roles in cell signaling and homeostasis (Valko et al., 2007). At low levels, ROS stimulates the synthesis of anti-oxidants (Poljsak et al., 2013); however, during ischemia or hypoxia, especially during reperfusion or reoxygenation, ROS production exceeds the scavenging and antioxidant defenses may be compromised and eventually overwhelmed, which can result in inflammation, apoptosis and heart dysfunction (Granger and Kvietys, 2015). High level of ROS activates the pro-apoptotic proteins caspase-3 and Bax, decreases the anti-apoptotic protein Bcl-2 (Ibanez et al., 2015), and initiates inflammatory response to ischemia/reperfusion injury (Granger and Kvietys, 2015) by increasing the inflammatory cytokines, IL-6, TLR-4, and TNR-α, etc. and decreasing anti-inflammatory cytokines (Yang et al., 2008; Kleinbongard et al., 2011; Liu et al., 2016b). Increase in the anti-inflammatory cytokine IL-10 has been found to be cardioprotective (Markowski et al., 2013). The present study showed that at the cellular level with hypoxia/reoxygenation insult, as in *ex vivo* hearts with ischemia/reperfusion injury (Liu et al., 2016b), acacetin down-regulated the increased pro-apoptotic proteins and pro-inflammatory cytokines and up-regulated the reduced anti-apoptotic proteins and anti-inflammatory proteins. These effects are clearly resulted from the inhibition of ROS over production induced by hypoxia/reoxygenation insult.

Our results showed that the inhibition of ROS production by acacetin is clearly related to the activation of Nrf2. It is well-recognized that Nrf2 is a key transcription factor that exists in all types of cells and regulates the expression of antioxidant pathway proteins that protect against oxidative damage triggered by tissue injury (Moi et al., 1994; Gold et al., 2012). Nrf2 is kept in the cytoplasm and associates with Kelch-like ECH-associated protein 1 (Keap1) which degrade Nrf2 by ubiquitination (Itoh et al., 1999). Nrf2 translocates into the nucleus where it binds to the DNA at the location of the antioxidant response element (ARE) and initiates transcription of many antioxidative genes (Itoh et al., 1997; Hirotsu et al., 2012). Activation of Nrf2 results in the induction of many cytoprotective proteins including HO-1, SOD1, and SOD2, etc. (Higdon et al., 2012; de Oliveira et al., 2017). This notion is supported by the present study, in which acacetin activates Nrf2, induces the expression of HO-1, SOD1, and SOD2 and exerts protection of cardiomyocytes against hypoxia/reoxygenation insult. SOD2 is a well-known mitochondrial antioxidant; therefore, the increase of SOD2 implies that mitochondrial protection by acacetin may be involved in the cardiomyocytes protection.

Nrf2 has been considered to be a drug target in the treatment of cardiomyocytes injury, cardiac dysfunction and other disorders (Li et al., 2011; Xing et al., 2012; Zhang et al., 2017). Nrf2 activators may ameliorate tissue injury induced by oxidative stress through preserving the antioxidant systems and reduce ROS production (Yoon et al., 2008; Wang L. et al., 2016; Xu et al., 2016). The present study provided the novel information that acacetin confers protection of cardiomyocytes against hypoxia/reoxygenation insult through activating Nrf2, since silencing Nrf2 removes the protection of acacetin against hypoxia/reoxygenation in H9C2 cardiomyoblasts. However, acacetin is not a direct activator of Nrf2 signal pathway.

Several natural compounds, e.g., quercetin (Li et al., 2016), danshensu (Hu et al., 2016), sulfuretin (Lee et al., 2014), and emodin (Park et al., 2016), have been reported to mediate Nrf2 activation by increasing the phosphorylation of P38, Akt, JNK and ERK1/2, and AMPK, respectively. In the present study, we demonstrated that acacetin at low concentrations of 1 and 3 μM did not increase the pP38, pAkt, pJNK or pERK1/2, but significantly increased phosphorylated AMPKα. Silencing AMPKα reduced Nrf2 expression and abolished Nrf2 activation by acacetin. In addition, in anesthetized rats, the Nrf2 activation was blocked by the AMPK inhibitor Compound C. These results strongly supports the notion that acacetin mediates Nrf2 activation through AMPK.

The observation that pAMPK is increased by acacetin is supported by a recent report in 3T3-L1 preadipocytes (Liou et al., 2017), in which acacetin inhibits adipocyte differentiation by activating pAMPKα; however, significant effects of acacetin on pAMPKα and adipo-differentation were only observed at high concentrations of 10–100 μM, not at therapeutic concentrations of 0.3–3 μM used in the present and previous study (Liu et al., 2016b). The difference in concentrations may be related to various tissue/cell types. In addition to quercetin (Li et al., 2016), danshensu (Hu et al., 2016), sulfuretin (Lee et al., 2014), and emodin (Park et al., 2016), xanthohumol, resveratrol, and berberine also mediate Nrf2 activation by stimulating pAMPK (Mo et al., 2014; Tamaki et al., 2014; Lv et al., 2017). Moreover, the flavanone butin has recently been found to be cardioprotective against ischemia/reperfusion injury in diabetic mice via AMPK/GSK-3β/Nrf2 signaling pathway (Duan et al., 2017). Although these natural compounds show promising therapeutic potential, their low solubility and low bioavailability are a barriers for drug development. Acacetin is a new member of AMPK activators and its water-soluble prodrug (Liu et al., 2016a,b) makes developing an injectable that can be used clinically to treat ischemic cardiac disorder in addition to atrial fibrillation highly feasible in the near future.

In this study, we did not explore how acacetin crosses the cell membrane to induce activation of the signaling pathways, how it interacts with any protein or receptor on the cell membrane, how much extracellular flavone enters into the cell, and how it is metabolized during ischemia exposure. However, these limitations would not affect the conclusion that acacetin protects cardiomyocytes against hypoxia/reoxygenation insult by activating a series of intracellular beneficial molecules.

Collectively, the present study provides the novel information that the natural flavone acacetin confers significant cardiomyocytes protection against hypoxia/reoxygenation insult and also ischemia/reperfusion injury via AMPK-mediated activation of Nrf2 signaling pathway. It may be a promising drug candidate that can be used for managing ischemic cardiac disorders in addition to atrial fibrillation.

## Author Contributions

W-YW, GL, YWu, YWang, and G-RL conceived and designed the experiments. W-YW, Y-DL, Y-KC, CW, and Y-XH performed the experiments. W-YW, L-JJ, and G-RL analyzed the data. W-YW and G-RL wrote the paper. All authors approved the submission.

## Conflict of Interest Statement

The authors declare that the research was conducted in the absence of any commercial or financial relationships that could be construed as a potential conflict of interest.
