# Supplementary material for: The Natural Flavone Acacetin Confers Cardiomyocyte Protection Against Hypoxia/Reoxygenation Injury via AMPK-Mediated Activation of Nrf2 Signaling Pathway
Source: Front Pharmacol. 2018 May 15;9:497. doi: 10.3389/fphar.2018.00497 (PMC5962741; doi:10.3389/fphar.2018.00497)
Supplement: Supplementary file 1 [file Presentation_1.pdf]

Supplementary Figures 1-3

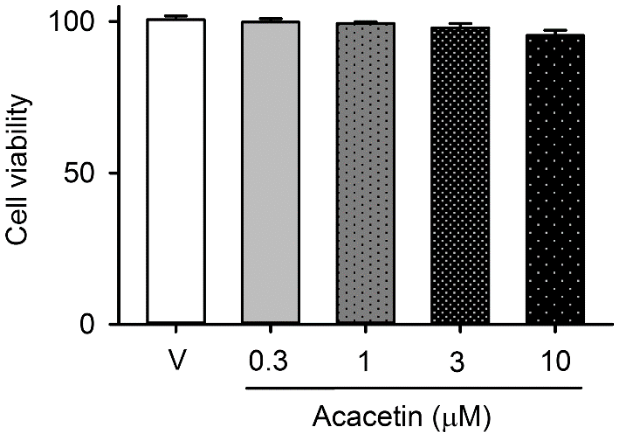

Figure S1. Effect of acacetin on cell viability. The cell viability was assayed by MTT in H9C2 cardiomyoblasts incubated without or with acacetin (0.3, 1, 3, or 10 μM) for 24 h. Acacetin had no effect on cell viability (n=5, mean± SEM).

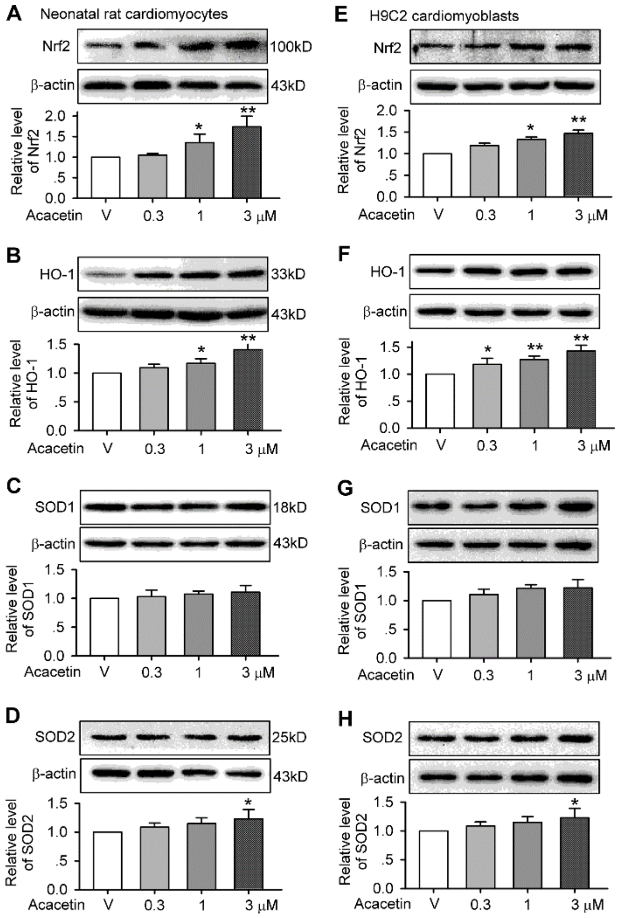

Figure S2. Effects of acacetin on antioxidative proteins. A-D. Western blots and mean relative levels of Nrf2 (A), HO-1 (B), SOD1 (C), SOD2 (D) in neonatal rat cardiomyocytes in the absence (V, vehicle) or presence of 0.3, 1 or 3 μM acacetin. **E-H.** Western blots and mean relative levels of Nrf2 (E), HO-1 (F), SOD1 (G), SOD2 (H) in H9C2 cardiomyoblasts in the absence and presence of 0.3, 1 or 3 μM acacetin. Data were expressed as mean ± SEM and analyzed by one-way ANOVA followed by Bonferroni-test (n=5 individual experiments, **P*<0.05, ***P*<0.01 vs. control).

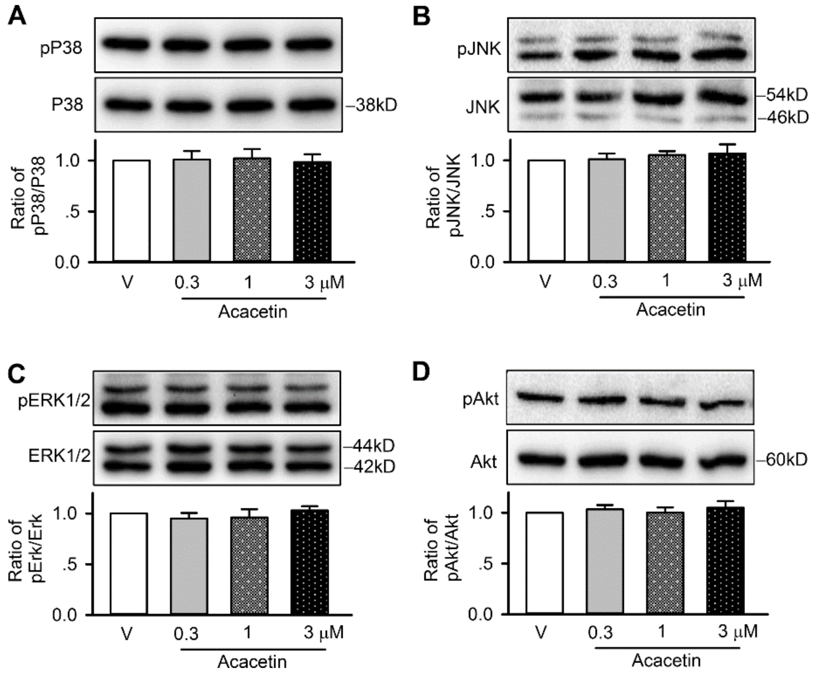

Figure S3. Acacetin has no effect on pP38 (A), pJNK (B), pERK1/2 (C), or pAkt (D) in H9C2 cardiomyoblasts (n=5, V, vehicle, mean ± SEM).
